# Gastrointestinal endoscopy training in the United States: Program structure and competence assessment

**DOI:** 10.1002/deo2.401

**Published:** 2024-07-07

**Authors:** Yutaka Tomizawa

**Affiliations:** ^1^ Department of Medicine Division of Gastroenterology University of Washington Seattle USA

**Keywords:** competency, credential, endoscopy, therapeutic endoscopy, training

## Abstract

The general principles of gastrointestinal endoscopy training in the United States were formulated and summarized more than a decade ago and the principles have been consistent until now. To summarize, trainees should be prepared to (i) appropriately recommend endoscopic procedures as indicated by the findings of the consultative evaluation, with an explicit understanding of accepted specific indications, contraindications, and diagnostic/therapeutic alternatives, (ii) perform procedures safely, completely, and expeditiously, including possessing a thorough understanding of the principles of conscious sedation/analgesia techniques, the use of anesthesia‐assisted sedation where appropriate, and pre‐procedure clinical assessment and patient monitoring, (iii) correctly interpret endoscopic findings and integrate them into medical or endoscopic therapy, (iv) identify risk factors for each procedure, understand how to minimize each, and recognize and appropriately manage complications when they occur, (v) acknowledge the limitations of endoscopic procedures and personal skills and know when to request help, and (vi) understand the principles of quality measurement and improvement. This article provides an overview of the endoscopy training system and structure, evaluation scheme, and competence and credentialing process in the United States.

## TRAINING PROGRAM

Trainees who learn and acquire endoscopic skills in the United States are primarily adult and pediatric gastrointestinal fellows (GI fellows) with limited exceptions including general surgery resident physicians in a certain training program environment.^1^ Fellowship training is advanced graduate medical education beyond a core residency program for physicians who desire to enter more specialized practice. Gastroenterology fellowship is the subspecialty training of internal medicine that focuses on the evaluation and treatment of diverse disorders of the GI tract, liver, pancreaticobiliary system, and nutrition. In this article, I will review and summarize the endoscopy training for adult GI fellows. GI fellows learn endoscopy as a part of their official GI fellowship training to become an independent gastroenterologist, therefore endoscopy training is incorporated into an official 3‐year GI fellowship training. On rare occasions overseas where there are certain training programs or even independent divisions of endoscopy (e.g., division of GI endoscopy), those programs or divisions do not exist in the United States. Training programs must be recognized and accredited by the Accreditation Council for Graduate Medical Education (ACGME). ACGME is a non‐profit private council that evaluates and accredits all graduate medical training programs (i.e., internships, residencies, and fellowships) for physicians in the United States.^2^ It is of note that ACGME is fully independent of certain professional organizations (e.g., the American Board of Internal Medicine [ABIM] or the American Society of Gastrointestinal Endoscopy [ASGE]). Physicians who successfully complete GI fellowship training will be eligible for the Gastroenterology Subspecialty Board of ABIM and will be board‐certified upon successful passage of the official board examination. GI endoscopy is one aspect of gastroenterology clinical practice, and board certification of GI endoscopy does not exist in the United States.

## TRAINING STRUCTURE

Competency‐based goals and objectives for each educational experience are designed to promote progress on a trajectory to autonomous practice in the field of gastroenterology. These must be distributed, reviewed, and available to GI fellows and faculty members. It is widely recognized consensus that gastroenterologists performing routine diagnostic and therapeutic endoscopy (e.g., control of GI bleeding [GIB]) require training to achieve basic and clinical knowledge, judgment skills, and the technical competence requisite for independently performing these studies in the GI fellowship training. All gastroenterologists should be skilled in the approach to the diagnosis and the endoscopic management of patients with GIB, including acute upper GIB of both variceal and nonvariceal origin and lower GIB of either acute or chronic presentation. Advanced endoscopic procedures, such as endoscopic retrograde cholangiopancreatography (ERCP), diagnostic and therapeutic endoscopic ultrasound (EUS), endoscopic mucosal resection, and placement of enteral stents, require additional training in advanced therapeutic endoscopy fellowship. Not all trainees can or should be offered comprehensive training in advanced therapeutic endoscopy. Furthermore, not all programs are capable of providing training in advanced therapeutic endoscopic procedures.

## TRAINING PROCESS

Endoscopic training takes place within the framework of clinical care and problem‐solving. GI fellows are exposed to a sufficient number and variety of common and uncommon disorders in the setting of both new and follow‐up inpatient and outpatient environments, which provide a broad endoscopic experience for GI fellows. In some programs, a certain period of time (usually a couple of months during the entire GI fellowship training) is dedicated to use only for outpatient endoscopic procedures, however, it does not apply to all GI fellowship training programs (Table 1). GI fellowship programs should provide experienced endoscopic supervisors who continually maintain and improve GI fellows’ abilities and possess the talents required to teach endoscopy. Initially, GI fellows may observe a few procedures performed by supervisors, followed by first attempting only diagnostic aspects of a procedure. At this stage, under constant direct supervision, GI fellows learn key principles of anatomy and basic scope manipulation such as esophageal and pyloric intubation and retroflexion of the scope tip. GI fellows will progress to performing the entire procedure and attempting therapeutic interventions including endoscopic clipping, hemostatic therapy in a bleeding patient, and polypectomy during colonoscopy. GI fellows are expected to progress through stages of decreasing supervision in which the trainees are deemed competent to complete a procedure with reasonable safety and patient comfort. Ultimately, GI fellows should reach a stage in which they are deemed competent by the endoscopic training director to perform a procedure without direct assistance and to recognize pertinent findings and address them when problems arise. The ACGME constraints however mandate that supervision be maintained for all procedures regardless of the GI fellows’ proficiency. GI fellows will then perform endoscopic procedures without supervision after a 3‐year of GI fellowship training. GI fellows also learn sedation techniques, integration of findings into a plan of endoscopic treatment, and develop skills for writing and appropriate documentation of endoscopic findings.

**TABLE 1:** Example of gastrointestinal (GI) fellowship training curriculum in the United States. This is the author's own experience.

GI fellow year‐1 (month)	GI fellow year‐2 (month)	GI fellow year‐3 (month)
GI inpatient consult (1)	GI inpatient consult (4)	GI inpatient consult (2)
Liver inpatient consult (2)	Liver inpatient consult (1)	Liver inpatient consult (1)
IBD in/outpatient (2)	Ambulatory (1)	IBD in/outpatient (1)
Pancreas/biliary (1)	Research (6)	Pancreas/biliary (1)
Nutrition (1)	Vacation (1)	Nutrition (1)
Endoscopy (2)		Research (6)
Ambulatory (1)		Vacation (1)
Research (2)		
Vacation (1)		
Continuity clinic ½ day per week throughout a year		

Abbreviations: GI, gastrointestinal; IBD, inflammatory bowel disease.

## STANDARD PROCEDURES

Standard procedures are readily available to most patients and therefore expected to be performed by any endoscopists. All GI fellows can expect to master these procedures during a 3‐year GI fellowship training period (including a minimum core period of 18 months of clinical training mandated by ACGME). Standard procedures include esophagogastroduodenoscopy, flexible sigmoidoscopy, colonoscopy, capsule endoscopy, mucosal biopsy, polypectomy, dilation of peptic strictures of the esophagus, and percutaneous endoscopic gastrostomy. Although the delivery of endoscopic hemostasis (injection and cautery techniques, esophageal variceal band ligation) requires considerable endoscopic expertise, mastery of these techniques is essential for every gastroenterologist performing endoscopy in bleeding patients and is thus included among standard procedures. Table 2 represents the threshold number of procedures that must be performed before competency can be assessed. The number represents a minimum, and it is understood that most trainees will require more (never less) than the stated number.^3^

**TABLE 2:** The threshold number of procedures for assessing competence.

Procedure	Required number
EGD	130
Including treatment of non‐variceal hemorrhage (10 actively bleeding)	25
Including treatment of variceal hemorrhage (five actively bleeding)	20
Esophageal dilation (guidewire and through the scope)	20
Colonoscopy	140
Including snare polypectomy and hemostasis	30
Percutaneous endoscopic gastrostomy placement	15
Capsule endoscopy (small bowel)	25

Abbreviation: EGD, esophagogastroduodenoscopy.

## ADVANCED PROCEDURES

GI fellows who elect to pursue a career as therapeutic endoscopists generally require an additional year (at a minimum of 12 months and 24 months at some training program/institution) of training beyond the standard 3‐year GI fellowship training focusing on therapeutic endoscopic procedures. Advanced procedures include but are not limited to ERCP, EUS, and all associated interventions, dilation of complex luminal strictures, enteral stent placement, Barrett's esophagus ablation therapies, endoscopic mucosal resection, balloon‐assisted enteroscopy, endoscopic tumor ablation, and so‐called third space endoscopy. In other words, those advanced procedures are not expected to be taught to all GI fellows. Advanced procedures are more complex and technically demanding to perform and often carry a relatively higher risk of diagnostic and therapeutic components, and technical skills for advanced endoscopic procedures must be acquired in a sequential fashion. Proficiency develops incrementally through the performance of sufficient numbers of procedures under direct supervision in a methodical sequence of increasing complexity.^4^ In certain circumstances, select advanced procedures (mainly ERCP) were used to be taught during the standard 3‐year training program however, it has gained consensus under the current healthcare environment and the nature of procedural complexity that concentrates advancing techniques in a relatively small number of highly trained individuals is ideal. Providing exposure to an advanced procedure such as ERCP during the standard 3‐year GI fellowship with the expectation that the trainee will subsequently complete training in practice is no longer appropriate in the United States. A guideline for training programs in advanced endoscopy was published by the ASGE in 1994. ASGE has been providing a formal match program in conjunction with participating advanced endoscopy training programs to make the process of application and interviewing fair and efficient for both the programs and applicants (GI fellows who want to pursue advanced endoscopy training).^5^

## ENDOSCOPY TRAINING DIRECTOR AND FACULTY

The ABIM has determined that specific methods for observation, evaluation, and documentation of procedural skills should be left to the discretion of the program directors (GI fellowship training director for general procedures, and advanced endoscopy fellowship director for advanced procedures). When GI fellows perform endoscopic procedures, all trainees should be observed regularly by the supervising faculty. As it is recommended by the ABIM, GI fellows keep documenting procedures they performed by a computer record or log book that identifies and evaluates the procedure(s) and any complications. This evaluation should become part of the trainees’ files. Programs offering advanced endoscopy training should have sufficient (a minimum of two) advanced endoscopists capable of performing and providing instruction in advanced procedures. Advanced endoscopy fellows should perform advanced procedures directly supervised by an experienced endoscopist (1:1 setting) knowledgeable in the indications for the procedure, the techniques of performing and the method of recording the results of the procedure, and the clinical significance of the findings. One of the advanced endoscopists takes the role of advanced endoscopy fellowship director and is fully responsible for curriculum creation and competency assessment for the trainee.

## COMPETENCY ASSESSMENT

The competency of all GI fellows should be documented by the program directors. The program directors have the responsibility of confirming or denying the technical competency and endoscopic exposure of trainees if warranted. Evaluation encompasses both cognitive and technical abilities. Trainee log books and records of procedural numbers should be provided by the GI fellows to the training director as a raw record of trainee experience. The threshold procedure numbers have been proposed by the ASGE training committee and the numbers may only be a rough benchmark for guiding trainee evaluation. Given available data, it suggests that at least 25–30 flexible sigmoidoscopies, 130 upper endoscopies, and 200 colonoscopies are required at a minimum before the trainee can be assessed for competence. Similarly, 180–200 ERCPs and 100 EUS are needed for acceptable accuracy for esophageal tumor staging; most experienced endoscopists agree that pancreatobiliary EUS demands more experience than esophageal EUS, whereas 40–50 cases may provide adequate preparation for the accurate evaluation of submucosal lesions.^1^ It should be noted that these threshold numbers represent a minimum benchmark before which a trainee's competency should not even be assessed. Most GI fellows will feel comfortable performing procedures independently and achieve competence much later. On the other hand, training programs must be able to meet and exceed these procedural volumes for each trainee. ASGE provides a core curriculum for a variety of endoscopic procedures and both trainer and trainee easily access their training self‐reference.^6^, ^7^, ^8^ ACGME announced the newer accreditation system in 2014 which focused on (i) ensuring that milestones are reached at various points in training, (ii) ensuring that competence is achieved by all trainees, and (iii) making certain that these assessments are documented by their programs. In response to meet the needs of accomplishing the tasks and the necessity of validated assessment tools, ASGE released two new evaluation tools for the assessment of competency of endoscopy for the core procedures of colonoscopy and esophagogastroduodendoscopy. These tools were designed by the ASGE training committee based on previously validated independent research for endoscopy competency assessment.^9^ The assessment tool can be used in multiple ways depending on how each training program has its endoscopy rotations structured and training directors tailor the methods used for form completion to fit their program structure and needs. Specific criteria for teaching directors or endoscopy faculty should follow do not exist, and the decision on whether GI fellows are granted to independently perform endoscopic procedures after the formal graduation is at the discretion of each training director or program. At my own institution, we randomly select a specific day when GI fellows rotate on out‐patient endoscopy procedure rotation and assess all endoscopic procedures performed on the day, and we assign a couple of assessment days at every level of training (i.e., the first‐year through the third year) before the final competency assessment.

## AFTER COMPLETION OF TRAINING AND CREDENTIALING

Fellowship (either gastroenterology or advanced endoscopy) graduation certificate will be provided by the program director, which certifies procedural competence for the trainees. Privileging to independently perform endoscopic procedures in the clinical setting after training occurs under the individual hospital credentialing committees. The aforementioned objective performance criteria were just as minimum standards for trainees and should serve as useful benchmarks for hospital credentialing authorities addressing this issue, and it is widely acknowledged most trainees will become more adept with additional experiences even after the fellowship training period. Although there are no established standards for monitoring ongoing procedural competence after the completion of training, self‐motivated initiative is a prerequisite and otherwise maintenance of expert performance cannot be assumed nor secured. Acquisition and maintenance of documented levels of competency in the skills of endoscopy in the GI fellowship and advanced endoscopy training have important and positive implications for the quality of patient care.

## CONFLICT OF INTEREST STATEMENT

Yutaka Tomizawa serves as a consultant for Boston Scientific Company, Medtronic, and AI Medical Service Inc.

## ETHICS STATEMENT

‐Approval of the research protocol by an Institutional Reviewer Board: N/A.

‐Informed consent: N/A.

‐Registry and the registration no. of the study/trial: N/A.

‐Animal studies: N/A.
